# 146 The vAdBeCeDa® program improves postural stability in non-visual older adults living in the community

**DOI:** 10.1093/eurpub/ckae114.192

**Published:** 2024-09-26

**Authors:** Katja Tomazin, Zala Zupan, Tjasa Knific, Maja Dolenc

**Affiliations:** University of Ljubljana, Faculty of Sport, Ljubljana, Slovenia; University of Ljubljana, Faculty of Sport, Ljubljana, Slovenia; National institute for public health, Ljubljana, Slovenija; University of Ljubljana, Faculty of Sport, Ljubljana, Slovenia

## Abstract

**Purpose:**

The vAdBeCeDa® program is a novel multicomponent exercise program for the prevention of falls and frailty in community-dwelling older adults by improving strength, stability, balance, flexibility and walking speed through targeted exercises. The aim of the study was to investigate how vAdBeCeDa® affects postural performance under non-visual conditions.

**Methods:**

Forty-three community-dwelling older adults were recruited (age: 72.3±5.3 years, height: 166.1±12.5; mass: 75.9±15.0 cm). The Balance Error Scoring System (BESS) was used to assess postural performance under non-visual conditions. The BESS was performed in three stances (two-legged, tandem and one-legged) and under two conditions (firm and foam) without vison. In each posture, the observers counted the errors in deviations from the correct posture. Postural control under visual conditions was assessed with a one-legged stance on firm ground. All measurements were taken at the beginning of the study, 8 weeks later (control condition) and 16 weeks later (after 8 weeks of the vAdBeCeDa® program). The vAdBeCeDa® program was performed twice a week, with each session lasting 60 minutes. The exercises were designed to train strength, flexibility, aerobic fitness, balance and coordination (all exercises were designed in 4 levels so that the right intensity was achieved for all participants).

**Results:**

The preliminary results show that the vAdBeCeDa® program is a promising tool to improve postural performance under visual and non-visual conditions.

**Discussion:**

Vision impairments are commonly observed in older adults; therefore, the vAdBeCeDa® program appears to efficiently improve postural stability when older adults need to redistribute sensory information required for postural control from visual to somatosensory and/or vestibular cues.

**Support/Funding Source:**

The project is funded by the Ministry of Health of the Republic of Slovenia and the European Union – NextGenerationEU. The project is implemented in accordance with the plan under the development area: Health and Social Security, Component 1: Health (C4 K1), Investment: Strengthening the competences of health care staff to ensure quality of care, Project: Integration of Geriatric Care for the Elderly.

